# Laparoscopic and endoscopic cooperative surgery for cholecystogastric fistula: A case report

**DOI:** 10.1016/j.ijscr.2020.04.100

**Published:** 2020-05-15

**Authors:** Goshi Fujimoto

**Affiliations:** Department of Gastroenterological Surgery, Ofuna Chuo Hospital, 6-2-24, Ofuna, Kamakura, Kanagawa 247-0056, Japan

**Keywords:** CBD, common bile duct, CEF, cholecystoenteric fistula, CGF, cholecystogastric fistula, LECS, laparoscopic and endoscopic cooperative surgery, CLEAN-NET, combination of laparoscopic and endoscopic approaches to neoplasia with non-exposure technique, CT, computed tomography, ERCP, endoscopic retrograde cholangiopancreatography, PS, performance status, Laparoscopic and endoscopic cooperative surgery, LECS, Cholecystogastric fistula, Malignancy, Case report

## Abstract

**Introduction:**

Cholecystoenteric fistula (CEF) is rare in biliary tract surgery, and cholecystogastric fistula (CGF) is the rarest form of CEF. Although open cholecystectomy with the closure of the fistula is the gold standard treatment for nonobstructing biliary-enteric fistulas, the optimal treatment for CGF has not been established. Laparoscopic and endoscopic cooperative surgery (LECS), a minimally invasive surgery for gastric submucosal tumors, reportedly helps achieve favorable postoperative outcomes. This report presents a case wherein CGF was treated with LECS.

**Case presentation:**

An-84-year-old man with a history of chemotherapy for ileocecal diffuse large B cell lymphoma presented with fever and abdominal pain. He was diagnosed with cholangitis, and endoscopic lithotripsy was performed. Abdominal contrast-enhanced computed tomography revealed gastric wall thickening close to the gallbladder, indicating a malignant lymphoma. Esophagogastroduodenoscopy revealed a concavity on the anterior wall of the antrum of the stomach. Direct endoscopic cholangiography, which was performed by the injection of a contrast medium into the concavity, confirmed the concavity to be a CGF. Cholecystectomy with the closure of the fistula using the LECS was performed to check for suture line leakage and the positional relation of the suture line and the pylorus. The resected specimen showed acute and chronic cholecystitis without malignancy. The patient did not experience postoperative complications in the subsequent 3 months.

**Discussion:**

Resection and closure of the fistula using LECS were performed successfully.

**Conclusion:**

LECS can be performed for CGF. LECS enables intraoperative observation of the fistula and suture line, and thus reduces postoperative complications.

## Highlights

• Cholecystogastric fistula (CGF) is rarest form of cholecystoenteric fistula (CEF).
• Our patient had a CGF close to the pylorus.
• Laparoscopic and endoscopic cooperative surgery (LECS) was used to treat CGF.
• Intraoperative endoscopy helped see the margin between fistula suture and pylorus.
• LECS is a viable option to treat CGF, a rare CEF in biliary tract surgery.

## 1 Introduction

Cholecystoenteric fistula (CEF) is rare in biliary tract surgery; cholecystogastric fistula (CGF) is the rarest form of CEF [1]. Although the gold standard treatment for non-obstructing biliary-enteric fistulas is open cholecystectomy with the closure of the fistula; laparoscopic surgery has been performed in some cases [[2], [3], [4]]. Some reports describe the treatment of gastric submucosal tumors, such as gastrointestinal stromal tumors, using laparoscopic and endoscopic cooperative surgery (LECS). Various types of modified LECS procedure, including inverted LECS, non-exposed endoscopic wall-invasion surgery (NEWS), a combination of laparoscopic and endoscopic approach to neoplasia with non-exposure technique (CLEAN-NET), and closed laparoscopic and endoscopic cooperative surgery (closed LECS), have been reported [5,6]. Our report highlights a case of LECS in a patient with CGF. LECS enabled the precise intraoperative observation of the fistula and suture line. This work was reported in line with the SCARE criteria [7].

## 2 Case presentation

**Fig. 1:**
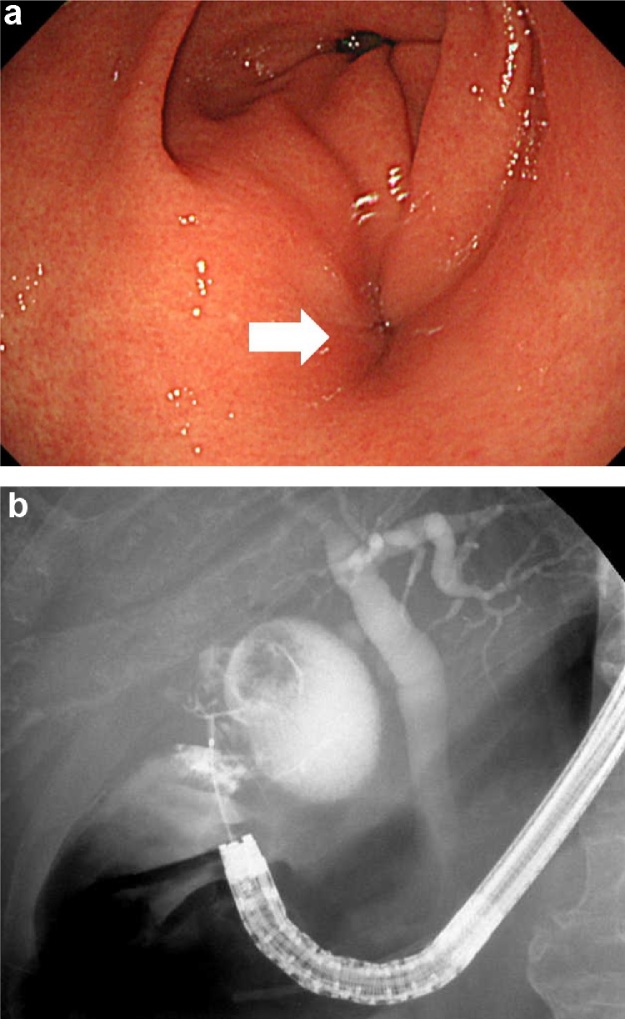
a. Findings on esophagogastroduodenoscopy. Esophagogastroduodenoscopy revealed a concavity (arrow) on the anterior antral wall of the stomach, which indicates a fistula. b. Endoscopic cholangiography findings of the fistula. Gastrografin injected from the concavity flowed into the gallbladder, which confirmed the diagnosis of cholecystogastric fistula.

An-84-year-old man with a history of chemotherapy for ileocecal diffuse large B cell lymphoma diagnosis with complete remission and free from chemotherapy, presented with fever and abdominal pain. His body mass index was 21.1 kg/m^2^ and his body temperature was 38.5 °C. His Eastern Cooperative Oncology Group Performance Status (PS) score was 0. Blood examination showed elevated levels of hepatobiliary enzymes in the serum. Abdominal ultrasonography showed a thickened gallbladder wall with cholelithiasis and pneumobilia of the intrahepatic bile duct. Endoscopic lithotripsy with endoscopic papillary balloon dilation and antibiotic therapy was used for the treatment of acute cholangitis, and this required hospitalization for 2 weeks. Endoscopic sphincterotomy and brush cytology were not performed. A thickened gastric wall close to the gallbladder, which indicated malignant lymphoma, was detected on computed tomography (CT) scan performed during hospitalization. Tumor markers in serum, including carcinoma antigen 19-9, carcinoembryonic antigen, and interleukin-2 receptor, were not elevated. Esophagogastroduodenoscopy showed a concavity on the anterior wall of the gastric antrum (Fig. 1a). Gastrografin injected from the concavity flowed into the gallbladder, which confirmed the diagnosis of CGF (Fig. 1b). The gastric wall thickening was presumed to be due to the inflammation associated with CGF. There were no malignant findings on the mucosa around the concavity, and no biopsy was taken. As the fistula was located near the pylorus, there was a risk of postoperative pyloric stenosis, and LECS was planned to check the positional relation of the suture line and the pylorus. The patient requested laparoscopic surgery and consented that since open surgery is the gold standard treatment for non-obstructing CEF, the procedure may be converted to open surgery if necessary.

**Fig. 2:**
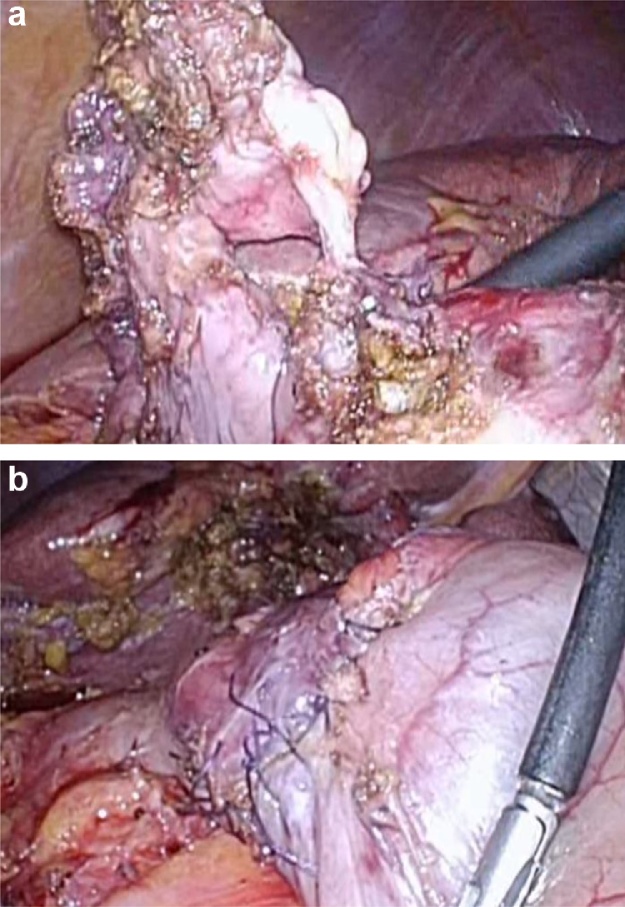
a. Findings of the fistula during laparoscopy. The fistula between the gallbladder and the stomach was exposed. b. Findings of the intraabdominal suture during laparoscopy. The dissected stomach wall of the fistular region was closed with an intraabdominal suture.

A camera port (Kii Balloon Blunt Tip System 12 × 100 mm) was inserted into the umbilicus via an open technique. Three additional ports (three 5-mm ports [Kii Access System 5 mm]) were inserted into the epigastric, right hypochondriac, and right upper quadrants of the abdomen with visual laparoscopic assistance under 10-mmHg pneumoperitoneum. A thick-walled contracted gallbladder was seen to be tightly attached to the omentum and the anterior wall of the gastric antrum. Because the bed side of the gallbladder wall was embedded in the liver by extensive inflammatory adhesions, partial cholecystectomy was performed, leaving the bed side of the gallbladder wall in place. After separation of the gallbladder from the liver, the cystic duct and cystic artery were cut. Subsequently, it became easy to observe the fistular region. The fistula was observed via endoscopy and marked with the injection of indigo carmine (1 mL of indigo carmine, 20 mL of 0.4% hyaluronic acid, and 20 mL of normal saline) into the submucosal layer around the fistula. Branches of the right gastroepiploic vessels in the excision area around the fistula were cut using an ultrasonically activated device (Harmonic Ace Plus; Johnson & Johnson, Tokyo, Japan). Adhesions between the gallbladder and stomach were dissected, and the fistula was exposed (Fig. 2a). Complete excision of the fistula was attempted to avoid postoperative perforation associated with unhealthy inflammatory tissue or the ischemic gallbladder wall, which was left on the stomach. After seromuscular dissection around the fistula, a pull-up and resection of the lifted mucosa that was surrounding the fistula was attempted using a linear stapler (Endo GIA™ Tristapler Purple 60 mm [Covidien]), via a combination of laparoscopic and endoscopic approaches to the neoplasia with a non-exposure technique (CLEAN-NET), which can avoid contamination of the gastric juice in the peritoneal cavity by the continuity of the mucosal layer. However, the thick-walled stomach made resection with an optimal margin from the pylorus difficult. Therefore, coring-out of the stomach wall around the fistular region was performed with an observation of the resection line laparoscopically and endoscopically, and the defect of the gastric wall was closed using intraabdominal hand-sewn sutures (Fig. 2b). After observing the suture line endoscopically, it was confirmed that the margins from the pylorus were enough. The absence of bleeding and air leaks was also confirmed by endoscopic insufflation, both endoscopically and laparoscopically. The total operative time was 4 h and 59 min, and the total intraoperative blood loss was 30 mL.

The resected specimen showed a thick-walled contracted gallbladder with gallstones measuring about 2 cm in diameter. Histologic examination revealed pseudo-pyloric gland metaplasia and Rokitansky-Aschoff sinuses. Thus, the patient was diagnosed with acute and chronic cholecystitis without malignancy. The patient did not experience postoperative complications in the 3 subsequent months.

## 3 Discussion

CEF is reported in 0.74% of patients undergoing biliary tract surgery [1] and 0.27–0.5% of patients undergoing laparoscopic cholecystectomy [3,4]. Cholecystoduodenal fistula is the most common form of CEF, occurring in 53% of cases; CGF is the rarest form of CEF, occurring in 2.1–4.2% of cases [1,3]. On the gallbladder side, the fundus was the fistula site in 19%, the body in 70%, and the Hartmann pouch in 11% of patients with concomitant CEF [4]. The fistular orifice in the stomach in patients with CGF is always at or near the pylorus [3,[8], [9], [10]]. Gallstone disease causes 91–94% of spontaneous internal biliary fistulas [1]. Local ulceration, pressure necrosis, and gradual erosion of intervening tissues resulting from the presence of a gallstone cause fistula of CEF [6,9]. Cancer, trauma, amebic infections, peptic ulcers, hydatid disease, and diverticulitis but not gallstone disease have been reported as causes of CEF [5,11].

A history of right hypochondriac or epigastric pain is the most common presentation besides jaundice, fever, anorexia, nausea, and vomiting [3,4]. Cholangitis is a common complication of CGF (40%), and the presence of pneumobilia might enable preoperative diagnosis of CEF [4]. CEF should be suspected in cases where cholecystolithiasis has lasted for over 5 years [3].

Preoperative examinations include ultrasonography, CT, magnetic resonance imaging, and endoscopic retrograde cholangiopancreatography (ERCP) [2]. Edema of the stomach wall and thickening of the gallbladder wall can be observed on CT scan. Gas in the gallbladder, adjacent to the stomach, suggests the presence of a fistula [9]. Although ERCP is reportedly the gold standard modality for the diagnosis of biliary fistula, its invasiveness should be considered. Furthermore, ERCP is unable to diagnose incomplete fistulas, which are closed on the inside and do not allow the contrast agent to pass [1,3]. The fistula of this patient could be detected directly by the injection of a contrast medium; this method seems useful when the fistular orifice can be detected via endoscopy. In cases of Mirizzi Syndrome, which accounts for 7.9% of CEF cases, a frozen section biopsy from the removed gallbladder can be examined for malignancy [4,12].

Since there are some reports of conservative treatment [10,11,13] or stone removal alone [8,14] for CEF, these less invasive treatments may be suitable in patients with a poor physical status. Although large balloon dilation may be effective for the treatment of common bile duct (CBD) stones, the recurrence rate of CBD stones is reported to be 34.1% at 2 years of follow-up [15]. The CBD stone recurrence rate of endoscopic sphincterotomy followed by endoscopic papillary balloon dilation is reportedly similar to that of endoscopic sphincterotomy alone; the recurrence rate of endoscopic sphincterotomy followed by endoscopic papillary balloon dilation is 6.9% at 13 months of follow-up [16]. Given the bacterial factors that cause the brown pigmented stones associated with cholangitis, CEF is at an increased risk of cholangitis if the fistula persists [17]. In our case, the patient is elderly, and stone removal with endoscopic sphincterotomy alone may be suitable. However, considering the good PS of the patient for his age, a high risk of CBD stone recurrence leading to cholangitis, and the fact that the life expectancy of 85-year-old Japanese men is up by 6.35 years, surgical treatment is thought to be acceptable.

Cholecystoduodenal fistula closes spontaneously in many patients [14]. However, the gold standard treatment for non-obstructing CEF should be open cholecystectomy with the closure of the fistula [2]. In some reports, CEF, gallbladder malignancy, and anesthetic risk were contraindications to laparoscopic cholecystectomy, and laparotomy was recommended when a cholecystocolonic fistula was detected incidentally during a routine laparoscopic cholecystectomy [2]. However, the benefits of laparoscopic surgery are increasingly reported in the literature [[2], [3], [4]]. Laparoscopic surgery for CEF resulted in shorter hospital stay, shorter operative time, less blood loss, less complication, and less mortality than did open surgery [3].

Regarding the intraoperative findings, dense inflammatory adhesions around the gallbladder, a shrunken and fibrotic gallbladder, which was firmly stuck to the adjoining viscera, and dense omental adhesions indicate the presence of CEF [3,4]; these findings were also seen in our case. The principle of laparoscopic management for CEF is the removal of the gallbladder and closure of the fistula [3]. Complete resection of the gallbladder wall is important to prevent postoperative perforation of the ischemic gallbladder wall and ensure that malignant tissue has not been retained. LECS facilitated complete resection of the gallbladder wall and a minimal resection range. Using a stapler in the transection of a fistula helps avoid contamination of the peritoneal cavity; however, a procedure via CLEAN-NET could not be chosen because of the thickened and fibrotic gastric wall [4]. The gastric suture line can be checked by air insufflation through a nasogastric tube during laparoscopic surgery for the treatment of CGF. In LECS, suture line leakage and the positional relation of suture line to the pylorus can be checked laparoscopically and endoscopically, which is useful to assess the risk of gastric outlet syndrome.

## 4 Conclusion

Laparoscopic cholecystectomy and fistula closure for CGF could be performed safely, even with the fistula located near the pylorus. LECS is useful for intraoperative observation of the fistula and suture line, which reduces postoperative complications.

## Patient perspective

The patient was concerned whether the surgery would convert to an open cholecystectomy and fistula closure, which requires a large incision. Therefore, he consented to this laparoscopic and endoscopic cooperative method to avoid the risk of a gastric outlet syndrome. There were no postoperative complications. The patient was informed about the regular medical follow-up.

## Declaration of Competing Interest

None.

## Sources of funding

This research did not receive any specific grant from funding agencies in the public, commercial, or not-for-profit sectors.

## Ethics approval

This case report was approved by the Research Ethics Committee of the Ofuna Chuo Hospital (No. 2019-005).

## Consent

Written informed consent was obtained from the patient for publication of this case report and the associated images.

## Author contribution

Goshi Fujimoto: Responsible for performing the procedure described in the case report, concept and design of the study, acquisition of data, drafting the manuscript, revising the manuscript, and approving the final version of the manuscript.

## Registration of research studies

This study was registered as a case report in the UMIN Clinical Trials Registry (https://www.umin.ac.jp/ctr/) with the unique identifying number UMIN000039775.

## Guarantor

Goshi Fujimoto.

## Provenance and peer review

Not commissioned, externally peer-reviewed.

## Availability of data and materials

The datasets supporting the conclusions of this article are included within the article.
